# Quantification of surface charging memory effect in ionization wave dynamics

**DOI:** 10.1038/s41598-022-04914-8

**Published:** 2022-01-21

**Authors:** Pedro Viegas, Elmar Slikboer, Zdenek Bonaventura, Enric Garcia-Caurel, Olivier Guaitella, Ana Sobota, Anne Bourdon

**Affiliations:** 1 Department of Physical Electronics, Faculty of Science, Masaryk University, Brno, Czech Republic; 2 Laboratoire de Physique des Plasmas (LPP), CNRS, Sorbonne Université, Université Paris Saclay, École Polytechnique, Institut Polytechnique de Paris, 91128 Palaiseau, France; 3 Department of Electrical Engineering and Electronics, Centre for Plasma Microbiology, the University of Liverpool, Brownlow Hill, Liverpool, L69 3GJ UK; 4 Laboratoire de Physique des Interfaces et des Couches Minces (LPICM), CNRS, École Polytechnique, Institut Polytechnique de Paris, 91120 Palaiseau, France; 5 Department of Applied Physics, EPG, Eindhoven University of Technology, Eindhoven, The Netherlands

**Keywords:** Plasma physics, Surfaces, interfaces and thin films

## Abstract

The dynamics of ionization waves (IWs) in atmospheric pressure discharges is fundamentally determined by the electric polarity (positive or negative) at which they are generated and by the presence of memory effects, i.e. leftover charges and reactive species that influence subsequent IWs. This work examines and compares positive and negative IWs in pulsed plasma jets (1 $\upmu$s on-time), showing the difference in their nature and the different resulting interaction with a dielectric BSO target. For the first time, it is shown that a surface charging memory effect is produced, i.e. that a significant amount of surface charges and electric field remain in the target in between discharge pulses (200 $\upmu$s off-time). This memory effect directly impacts IW dynamics and is especially important when using negative electric polarity. The results suggest that the remainder of surface charges is due to the lack of charged particles in the plasma near the target, which avoids a full neutralization of the target. This demonstration and the quantification of the memory effect are possible for the first time by using an unique approach, assessing the electric field inside a dielectric material through the combination of an advanced experimental technique called Mueller polarimetry and state-of-the-art numerical simulations.

## Introduction

Natural phenomena of atmospheric discharges, such as lightning and sprites developing above clouds, are initiated by plasmas propagating as ionization waves (IWs), characterized by either positive or negative streamer propagation mechanisms^1–5^. In fact, most lightning is negatively-charged but positive lightning also takes place, although with a much lower frequency in nature, of only around 10%^6^. IWs are not only precursors to high altitude discharges, but also characterize many laboratory plasmas at atmospheric pressure. An IW, or streamer, propagates thanks to a non-linear effect in its front, where local charge separation leads to a highly enhanced electric field driving ionization and propagation^7–9^. The polarity of the discharge ignition determines whether the front of the IW is charged negatively or positively and thereby whether negative or positive charges are attracted or repulsed. The polarity fundamentally determines the discharge morphology and dynamics, as well as its interaction with surfaces^10–12^.

When the propagation of IWs is limited by the presence of dielectric surfaces, the plasma is characterized as a dielectric barrier discharge (DBD), that allows to generate a highly reactive medium at low temperature and atmospheric pressure^13–15^. Ever since DBDs are studied, the true nature of their operation has not been fully described, for lack of appropriate diagnostics. By indirect evidence, the existence of memory effects, i.e. leftover charges and reactive species that influence subsequent discharges, has been stipulated. In fact, most features of DBD operation, from ignition to reproducibility, uniformity and jittering, rely on the general idea of memory effects. These can be present in the gas phase volume^16–18^ or on the dielectric surfaces^19^. In the recent work by Fan et al.^19^, a “wall voltage” in between repetitive discharges has been indirectly measured through a series of reference capacitors as a surface charging memory effect. In this work, we demonstrate and quantify a surface charging memory effect for the first time, by both directly measuring and simulating the spatial distribution of electric field inside a dielectric target impinged by pulsed plasma jets of different polarities. Furthermore, we show the concrete influence this memory effect has on discharge dynamics.

Plasma jets are very interesting for the study of interactions between low-temperature plasmas at atmospheric pressure and surfaces. In these devices, IWs propagate in a noble gas that flows through a dielectric tube and then expands into air, forming a plume, where the discharge interacts with target surfaces. As such, these tools repetitively deliver high electric fields and radiation to remote targets, as well as a wide range of reactive and charged species. Plasma jets therefore find applications in the areas of bio-medicine, agriculture and material processing, having raised a lot of attention in the last decades^20–25^.

One of the most interesting effects to study on plasma jet operation is the deposition of charges on the surface of dielectric targets and the generation of electric field inside the target materials, as these quantities are crucial both for applications and the study of memory effects. Particularly, the electric field produced by jets on targets is known to induce the apoptosis of cancer cells^26–29^. Moreover, surface charge distributions are important as a memory effect that may impact discharge dynamics and have an effect on flow dynamics. For instance, in Van Doremaele et al.^30^ the channelling of a He flow by positive and negative pulsed plasma jets has been attributed to electrohydrodynamic forces in long time-scales related to surface charges remaining on a glass target surface after the fall of the pulses. In that work it has been assumed that charges of the same sign as the polarity of applied voltage remain at the surface after the pulse. Usually, the surface charge and the electric field inside target materials are indirectly assessed^31^ or may be assumed^30^. Nevertheless, experimental methods have been developed to analyze electric field and surface charge density using electro-optic targets, whose refractive index changes linearly with the induced electric field^32,33^. In our previous works, we have shown that combining electric field measurements in He plasma jets with plasma simulations provides a highly reliable and detailed characterization of electric field and surface charge on targets^34–36^. However, these previous works have assessed positively pulsed jets and have not found direct evidence of surface charging memory effects.

In this work, we proceed with the study of charging of dielectric targets through simulations and experiments, addressing also pulsed jets of negative polarity. Comparing different polarities allows us to observe different distributions of leftover surface charges and electric fields remaining in the target long after the fall of the applied voltage pulse and in between pulses. Indeed, for positive polarity, negative charge deposition takes place after the pulse, mostly at the center of the target, and the target appears neutralized at longer timescales^35,36^, while for negative polarity the surface charging dynamics is so far less well known. Differences between jets operating with positive and negative polarity pulses have been identified in terms of discharge structure and dynamics in the tube^37–39^ and in the plume^12,40,41^, which is closely related to the fundamentally different discharge propagation mechanisms. As such, we can expect the target surface charging dynamics to also be strongly dependent on applied voltage pulse polarity.

In this article we show how the choice of applied voltage pulse affects the surface charging dynamics and the leftover charges and electric field in a BSO dielectric target impacted by a plasma jet. Rectangular pulses of applied voltage are studied, with approximately 50 ns rise-time and fall-time, around 1 $\upmu$s width and different amplitudes $V_\mathrm{P}=-5,-6,+5,+6$ kV. The temporal and spatial evolutions of the axial component of electric field inside the target are measured through Mueller polarimetry diagnostics. Simulations from a two-dimensional fluid model are used for comparisons with these measurements and to provide further information on discharge dynamics. First, the differences of discharge dynamics and interaction with the dielectric BSO target for the different pulses of applied voltage are presented. These reveal, for the first time, the direct influence of leftover surface charges as memory effect. Then, the surface charging dynamics leading to the different distributions of leftover surface charges and electric field is described, which provides an explanation for the different memory effects. Finally, the main conclusions are summarized. The experimental and numerical setups are described in the method sections, together with the specificities of the conditions for comparisons between experiments and simulations. This includes the description of how leftover surface charges are considered in simulations, why is pulse width slightly different in experiments and simulations and how to compare results in such conditions.

## Results

### The influence of polarity on plasma-target interaction leading to memory effects

**Figure 1:**
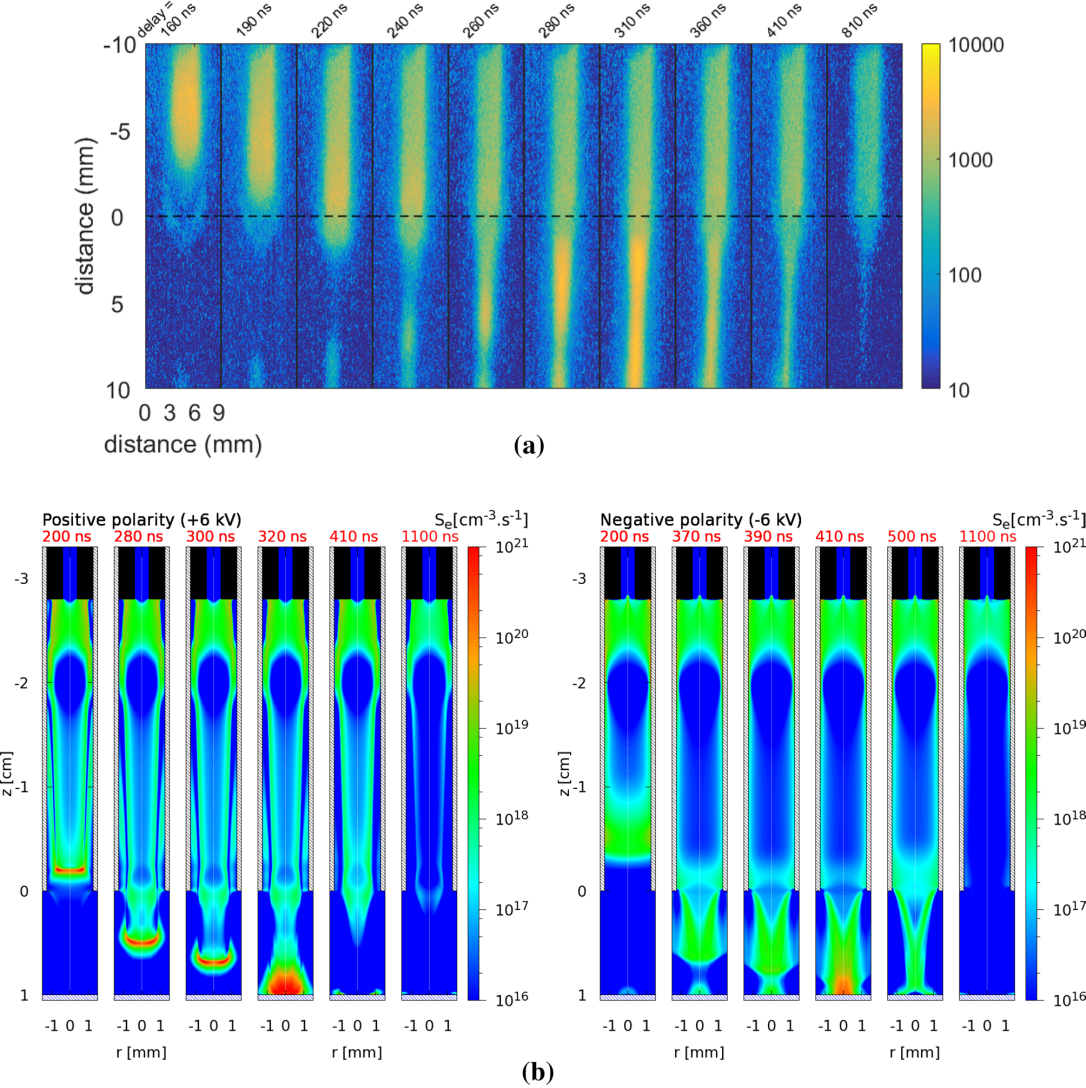
Dynamics of IW propagation during the pulse. (**a**) Experimentally-obtained imaging of light emission at different instants during discharge propagation and interaction with the BSO target, for $V_\mathrm{P} =-6$ kV. (**b**) Simulated spatial distribution of electron impact ionization source term ($S_\mathrm {e}$), during discharge propagation and interaction with the BSO target, for $|V_\mathrm{P}| = 6$ kV and both polarities of applied voltage. The instants in time represented in Fig. (**b**) refer to the time in simulations $t_\mathrm{s}$ and are not shifted. In both experimental and numerical setups, the outer ring electrode is located between $z=-2.3$ cm and $z=-2.0$ cm, the tube ends at $z=0$ and the target is placed at $z=1.0$ cm. Figure generated using Python 3.8^44^ and Gnuplot 5.0.2^45^.

The plasma-target interaction is preceded by the propagation of the IW and depends on the characteristics of its dynamics. Indeed, understanding the surface charging of the target requires a full depiction of the plasma acting as its precursor. The structure and dynamics of IW propagation during the pulse is represented in Fig. 1a from experimental imaging of light emission for $V_\mathrm{P} =-6$ kV. The discharge dynamics in this jet configuration has been studied for positive pulses in Viegas et al.^34^ and Slikboer et al.^35^. The experimental results for $V_\mathrm{P} =-6$ kV are compared with simulation results in Fig. 1b, for both $V_\mathrm{P} = +6$ kV and $V_\mathrm{P} = -6$ kV. In the simulations, initial surface charges are considered on the target, by taking the final surface charge distribution from a previous calculation, as explained in the Methods section. The spatial distribution of the electron impact ionization source term ($S_\mathrm {e}$) is represented in Fig. 1b, which can be qualitatively compared with experimental imaging of light emission, as has been done in previous works^34,42,43^. The same quantities for $|V_\mathrm{P}| = 5$ kV are represented in Supplementary Fig. 1 and briefly commented in Supplementary Discussion 1.

For negative polarity, Fig. 1 presents a qualitative agreement between experiments and simulations on discharge structure at the different stages of discharge dynamics. The IW propagates axially through the tube from the electrode region towards the end of the tube, reaching it approximately 200 ns after the start of the pulse. Then, discharge dynamics in the plume appears to be faster in experiments than in simulations. In both cases, the positive surface charge density on the center of the target surface is high enough (up to 25 nC cm$^{-2}$ in simulations, as will be shown later) to generate a high electric field and a relevant electron-impact ionization source term as the negative IW approaches. On the one hand, the presence of initial positive surface charges and associated electric field leads to the creation of a charged cloud on top of the target surface. On the other hand, as the target surface is globally negatively charged ( -505 pC, see Table 2), the electric potential difference between the inner electrode and the target is lower than in a scenario without initial surface charges and thus the negative IW propagation is slower. Simulations of negative IW propagation with initial surface charge have $\sim 30$ ns later impact than simulations in their absence. This is a demonstration of the surface charging memory effect and its direct influence on discharge dynamics. However, it appears that the charged cloud propagates more towards the incoming IW in the experimental case than in the simulations, leading to an earlier merge with the discharge in the experimental case.

For positive polarity of applied voltage, the discharge dynamics of propagation and interaction with the target has been characterized in our previous works^34–36,43^ for very similar cases. It should be noticed that, unlike for the case of negative polarity, the presence of the initial surface charges on the target in simulations has no influence on the IW dynamics of propagation for the positive polarity cases. As the initial negative surface charge density in the center of the target has magnitude below 10 nC cm$^{-2}$ in simulations, no charged cloud is generated in either experiments or simulations. Moreover, as the target is globally almost neutralized (total initial charge of  85 pC, see Table 2), the time of impact is delayed by only a few ns (less than 10 ns) with respect to simulations without initial surface charging. As such, the relevance of the surface charging memory effect appears to be dependent on applied voltage polarity.

Figure 1b shows that in simulations the velocity of propagation inside the tube is similar for the positive and negative discharges. However, the discharge structure in the tube is significantly different according to polarity, more homogeneous for negative discharge and more filamentary for positive discharge, as has been reported in experiments in He^38,39^ and in simulations in Ne–Xe^37^. Indeed, Fig. 1b shows that $S_\mathrm{e}$ has a clear maximum at the discharge front for positive polarity, while it is almost radially uniform in the negative case. This difference is closely related to the different discharge propagation mechanisms. For positive polarity, a radial sheath is created between the plasma bulk and the tube wall due to the limitation of outer electrons that can be pulled towards the plasma^46,47^, while in the negative case electrons are pushed outwards and the discharge can fill the space within the tube. The positive discharge mechanisms induce higher peak electric fields, $S_\mathrm {e}$ and electron densities than in the negative case^37^. Concerning IW propagation in the plume, Fig. 1b shows that in simulations the positive discharge is faster than the negative one, in agreement with other works in jet plumes^12,40,41^. Likewise, we find, as in experiments^40,41^ and in simulations^12^ in literature, that the IW front in the plasma plume has a more spherical form with positive polarity, while with negative polarity the shape of the discharge is like that of a sword. Finally, after the discharge impact on the target, slow radial spreading takes place on top of the target until the end of the pulse, for both polarities of applied voltage.

**Figure 2:**
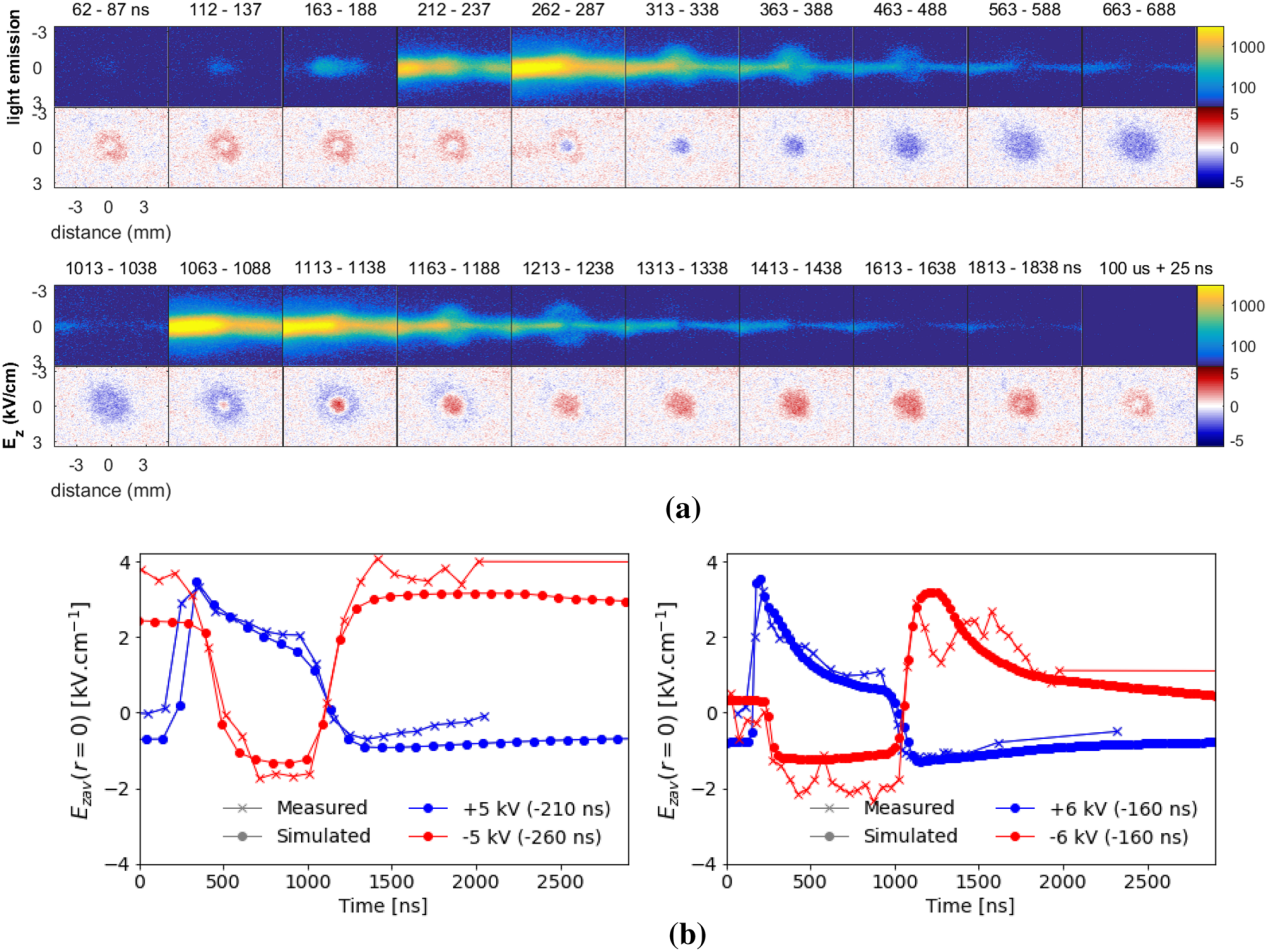
Surface charging during plasma-target interaction. (**a**) measured light emission and experimentally obtained axial electric field inside the BSO target at different instants, for $V_\mathrm{P} = -6$ kV. 25 ns averaging has been considered. (**b**) Temporal profiles of the electric field on the axis (at $r=0$) inside the BSO target, from simulations and measurements, for positive and negative polarities of applied voltage. On the left, for $V_\mathrm{P}=+5$ kV (ToI=293 ns and ToI$_\mathrm{s}$=480 ns) and $V_\mathrm{P}=-5$ kV (ToI=380 ns and ToI$_\mathrm{s}$=670 ns). On the right, for $V_\mathrm{P}=+6$ kV (ToI=193 ns and ToI$_\mathrm{s}$=320 ns) and $V_\mathrm{P}=-6$ kV (ToI=240 ns and ToI$_\mathrm{s}$=410 ns). The time considered in the figure is the one defined in experiments, as the simulation results have been shifted according to Table 2. Figure generated using Python 3.8^44^.

The plasma-target interaction both during the pulse and afterwards is investigated experimentally through the measurement of the spatial distribution, perpendicularly to the jet incidence, of the electric field in the axial direction ($E_\mathrm{z}$) inside the BSO target^34,35,48^. This is shown in Fig. 2a together with imaging measurements for $V_\mathrm{P}=-6$ kV at different instants during the pulse, shortly after the pulse and in between pulses at $t=100$ $\upmu$s (the period is of 200 $\upmu$s). The cases with positive applied voltage have been studied in detail in Slikboer et al.^35^ and the results for $V_\mathrm{P}=-5$ kV are shown in Supplementary Fig. 2. The instants in the figure correspond to the intervals during which the measurements are taken and averaged, with respect to the time $t_0 = 0$ when the pulse of applied voltage starts rising. We should notice that the electric field inside a dielectric target can be originated by charge separation in volume or deposited at the surface^34^, but in the case of a target of high permittivity, such as BSO ($\epsilon _\mathrm{r} = 56$), it results mostly from surface charges^36^.

Figure 2a shows that, after impact, charge deposition (electrons sticking on the surface) takes place on the target surface and the discharge spreads on top of the target. As such, the charging of the target depends on the charging time, i.e. the time during which the charging and spreading can take place, which is dependent on $|V_\mathrm{P}|$ and pulse width^35,36^. The electric field induced by the discharge in the target during the pulse is positive for $V_\mathrm{P}>0$^35^ and negative for $V_\mathrm{P}<0$, corresponding to positive and negative charging of the target, respectively. With positive polarity, the discharge on top of the target is subjected to some filamentation, producing star-shaped patterns of field inside the target^35^. Conversely, when the polarity is negative, the radial expansion of the field inside the target has a more uniform appearance, in agreement with the more diffuse character of negative discharge dynamics. As is the case for positive polarity^35^, the radial spreading of the negative discharge is faster and larger for higher $|V_\mathrm{P}|$.

For both polarities, as the pulse of applied voltage falls, an electric field of opposite sign to the one of the first discharge propagation is generated between the newly-grounded electrode and the charged plasma^43^. This electric field distribution propagates as a charge relaxation event that tends to neutralize the plasma channel, reported in many works and described in more detail for jets in^43^. In the current case, as this electric field redistribution impacts the BSO target, it leads to charge deposition of opposite sign (electron absorption for positive polarity and ion neutralization and electron emission for negative polarity), firstly in the center and then in a wider region, as represented by the electric field measurements inside the target in Fig. 2a. Then, in later times, charge deposition takes place slowly in the sense of neutralizing the surface charge on the target and thus the electric field inside the target tends to relax. However, while in the positive polarity cases the electric field inside the target in the long time-scale between pulses is negligible, it can be as high as +4 kV cm$^{-1}$ in the negative cases. For $V_\mathrm{P}=-6$ kV, the target is neutralized in the center, but positive field remains in the edges, while for $V_\mathrm{P}=-5$ kV the positive field remains also in the center, as shown in Supplementary Fig. 2. We can conclude that the sign, quantity and distribution of charges remaining in dielectric targets in between pulses is dependent on particular conditions and can challenge assumptions, such as those in van Doremaele et al.^30^, where it is considered that charges of the same sign as the polarity of applied voltage remain at the surface after the pulse.

The dynamics of surface charging of the target in simulations is very similar to the one described from experimental results. This is visible from the temporal evolution of electric field inside the target, at its center ($r=0$), averaged through its thickness ($E_{zav}$), presented in Fig. 2b from experimental and numerical results, for the different cases of applied voltage. The simulation results are averaged in time to have the same temporal resolution as measurements (100 ns averaging for $|V_\mathrm{P}|=5$ kV and 25 ns averaging for $|V_\mathrm{P}|=6$ kV). The influence of this averaging has been assessed in Viegas and Bourdon^36^ for positive polarity cases. As is the case for positive polarity^35^, the agreement between simulations and experiments is also excellent for $V_\mathrm{P}=-5$ kV and $V_\mathrm{P}=-6$ kV.

It is visible from Fig. 2b that, shortly after discharge impact on the target, $E_{zav}(r=0)$ reaches higher magnitude for positive than for negative polarity. Moreover, $|E_{zav}|(r=0)$ decreases after the impact at a lower rate for negative polarity than for the positive cases. This can be associated to the different radial spreading dynamics, that is faster and more filamentary for positive polarity. The less diffuse character of positive discharges leads to more intense and localized positive charging of the surface than in the negative case. In its turn, this leads to a faster compensation after the passage of the front, as the center of the target is partially neutralized. As the pulse of applied voltage falls, the center of the target is not only neutralized, but also charged by charges of opposite sign, generating an electric field inside the target in the opposite direction to the one present during the pulse. We can notice that, when this phenomenon takes place, the positive charging (for $V_\mathrm{P}=-5$ kV and $V_\mathrm{P}=-6$ kV) is also more intense and localized than the negative charging (for $V_\mathrm{P}=+5$ kV and $V_\mathrm{P}=+6$ kV). While for $V_\mathrm{P}>0$ the magnitude of this opposite polarity charging at the center increases with $|V_\mathrm{P}|$, it appears to decrease with $|V_\mathrm{P}|$ for $V_\mathrm{P}<0$. For later times, we see that the magnitude of the field falls at different rates according to the case of $V_\mathrm{P}$. The rate tendentiously decreases with time and, in the case of $V_\mathrm{P}=-5$ kV, $E_{zav}(r=0)$ appears to remain constant after the pulse. As the result of this dynamics, the surface charging memory effect, i.e. the leftover field between pulses inside the target at the point of discharge impact, depends on $V_\mathrm{P}$.

### Leftover charges on the target surface as memory effect

**Figure 3:**
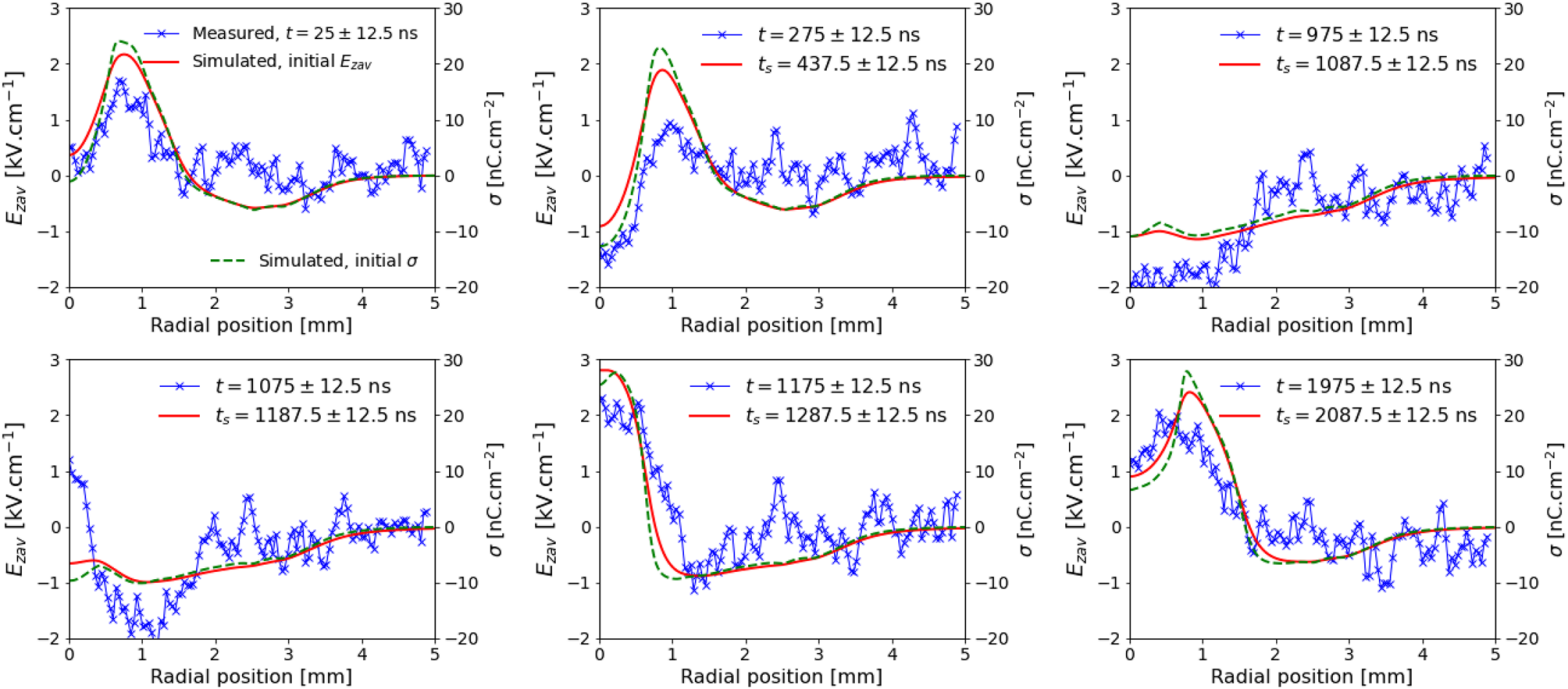
Radial profiles of surface charging for $V_\mathrm{P}=-6$ kV. Radial profiles at different instants of the axial component of electric field inside the BSO target ($E_{zav}$, solid lines) from simulations and measurements, and of the simulated surface charge density ($\sigma$, dashed lines) on the target surface, for $V_\mathrm{P}=-6$ kV. 25 ns temporal averaging is used. *t* refers to the instant in time in experiments and $t_\mathrm{s}$ to the instant in time in simulations. Figure generated using Python 3.8^44^.

To assess in more detail the dynamics of surface charges and their remainder in between pulses, Fig. 3 shows $E_{zav}(r)$ inside the BSO target at different instants, from simulations and measurements, for $V_\mathrm{P}=-6$ kV. The simulated surface charge density distribution $\sigma (r)$ is also represented, and is shown to have a similar radial profile as $E_{zav}(r)$, following a proportionality of approximately 10 nC cm$^{-2}$ to 1 kV cm$^{-1}$. This similarity is expected, since most of the electric field inside the target is generated by the surface charges^36^. These quantities are presented for $V_\mathrm{P}=-5$ kV in Supplementary Fig. 3. These quantities have been compared for a case with positive polarity in Slikboer et al.^35^, with excellent agreement between simulations and measurements. The instants for which these quantities are represented in this work are chosen as: initial, during discharge impact on the target, before the fall of the pulse, after the fall of the pulse, 100 ns later and 800 ns later. The simulation results in Fig. 3 are temporally-averaged, as in Fig. 2b.

**Figure 4:**
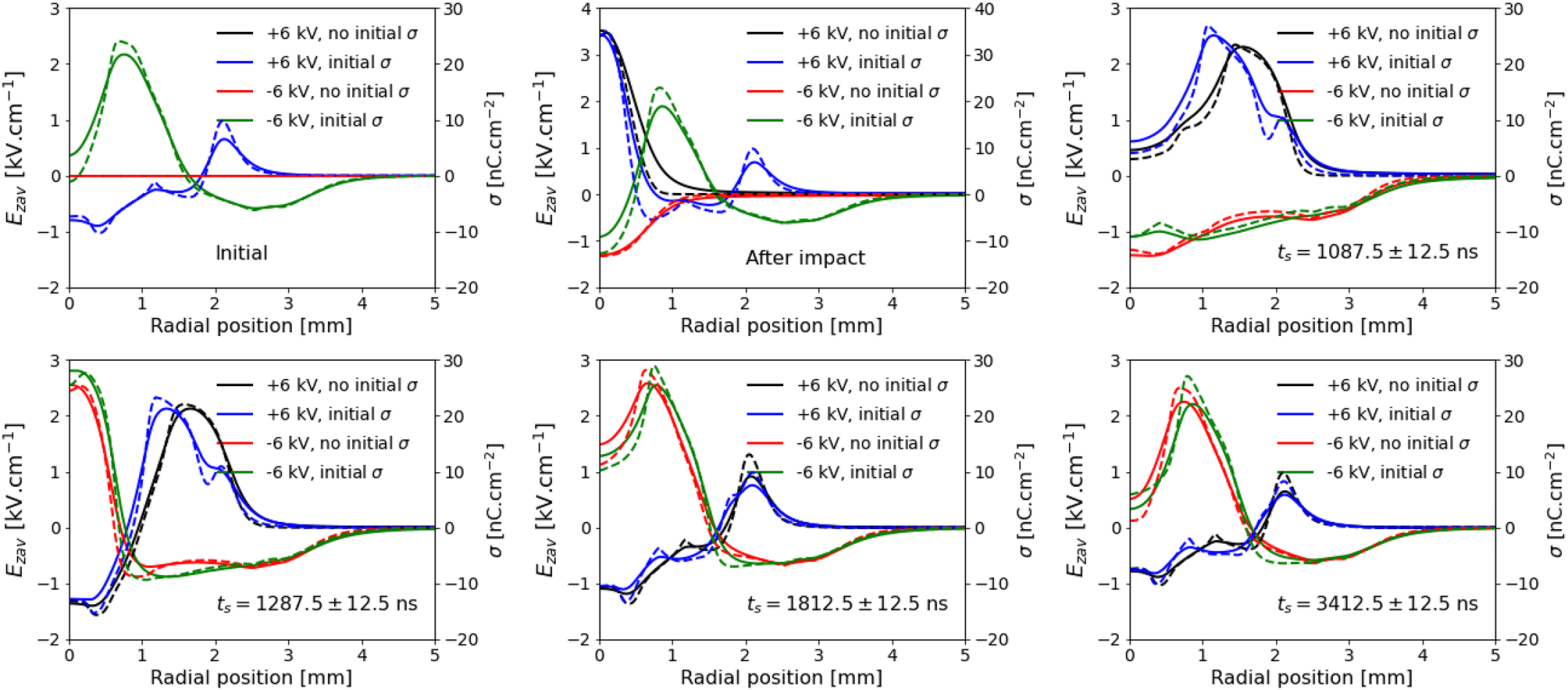
Simulated radial profiles of surface charging for $|V_\mathrm{P}|=6$ kV. Radial profiles at different instants of the axial component of electric field inside the BSO target ($E_{zav}$, solid lines) and of the surface charge density on the target surface ($\sigma$, dashed lines), from simulations, for $V_\mathrm{P}=-6$ kV and $V_\mathrm{P}=+6$ kV, with and without considering initial surface charges. 25 ns temporal averaging is used. Figure generated using Python 3.8^44^.

The agreement between simulations and experiments in Fig. 3 and in Supplementary Fig. 3 is excellent. The results clearly show that, for $V_\mathrm{P}=-6$ kV (Fig. 3), after the positive charge deposition that follows the end of the pulse, there is surface charge neutralization at the center of the target through electron absorption after $t = 1175$ ns. Conversely, for $V_\mathrm{P}=-5$ kV (Fig. 2b and Supplementary Fig. 3), there appears to be no absorption on the target surface after $t=1212.5$ ns. To complement the study of the temporal evolutions of the radial profiles of $E_{zav}$ and $\sigma$ in the target, a comparison is presented in Fig. 4 between the simulated cases with $V_\mathrm{P} = -6$ kV and $V_\mathrm{P} = +6$ kV, with and without considering initial surface charges on the target. Six instants in time are considered: initial, after the discharge impact, before the fall of the pulse, 200 ns later, 550 ns later and 1600 ns later. The same quantities are represented for $|V_\mathrm{P}| = 5$ kV in Supplementary Fig. 4.

In Fig. 4 and Supplementary Fig. 4, it is shown that the initial surface charges have an influence on the simulation results of $E_{zav}$ and $\sigma$ before discharge impact on the target and shortly after the impact. However, the results are already very similar at the end of the pulse at $t_\mathrm{f}=1100$ ns (1200 ns for $|V_\mathrm{P}| = 5$ kV) and are almost the same by the end of each simulation for the same $V_\mathrm{P}$. Moreover, the results confirm that the surface charging dynamics is strongly dependent on the polarity of applied voltage and that high leftover field remains inside the target after the pulse for the negative polarity case. For positive polarity, it is visible in Fig. 4 and in Supplementary Fig. 4 that the magnitude of the leftover field lies within the experimental errorbar of 1 kV cm$^{-1}$.

**Figure 5:**
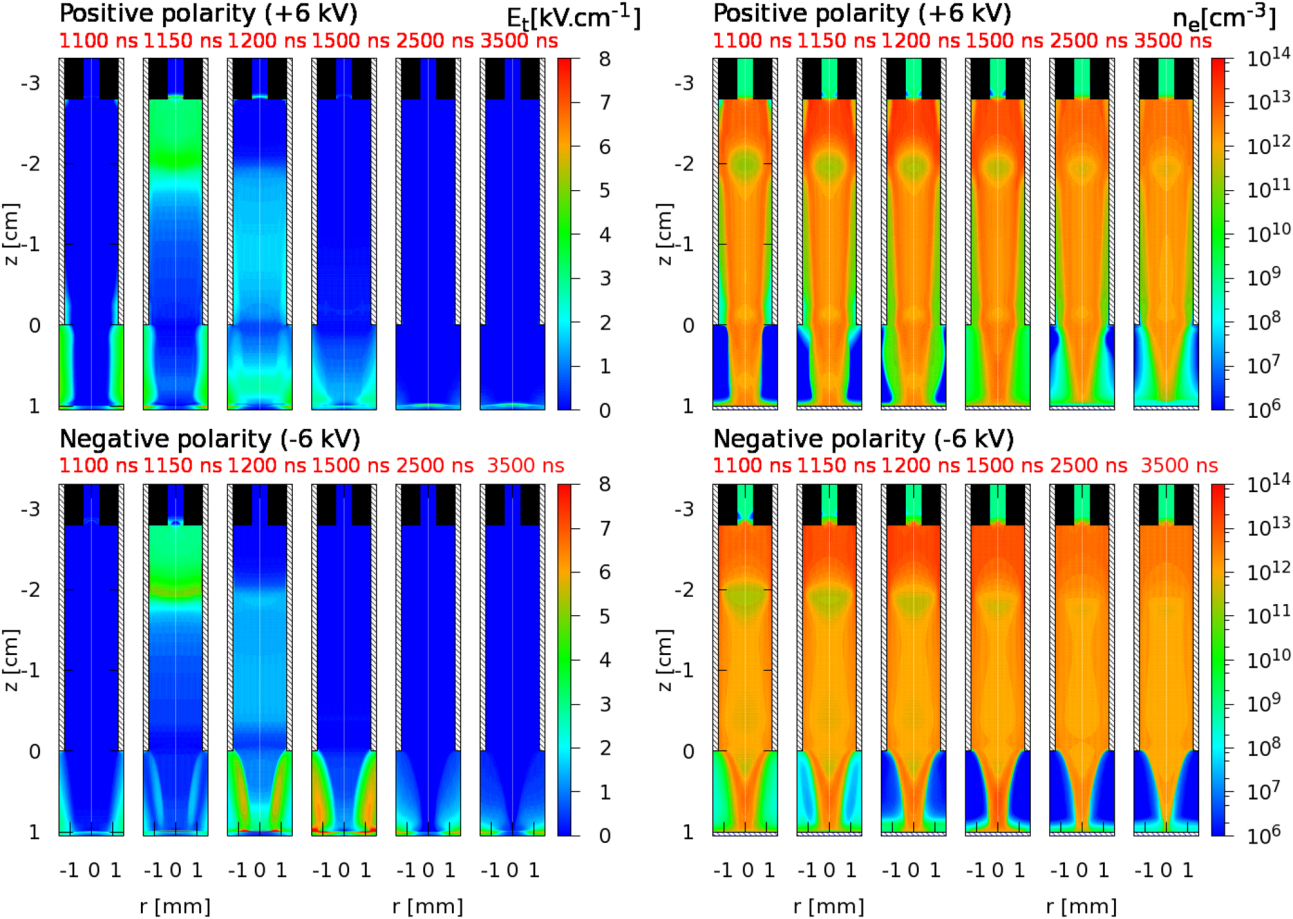
Simulated discharge dynamics after the pulse. Electric field magnitude ($E_\mathrm {t}$) and electron density ($n_\mathrm {e}$) distributions from simulations, at the fall of the pulse, for $|V_\mathrm{P}|=6$ kV and both polarities of applied voltage, at different instants $t_\mathrm{s}$. Figure generated using Gnuplot 5.0.2^45^.

Charges on the surface of the target generate an electric field not only inside the target but also in the plasma, attracting charges of opposite signal through electric drift. That explains why, during the pulse (between impact and $t_\mathrm{f}=1100$ ns in the case of $|V_\mathrm{P}|=6$ kV) and especially shortly after the fall of the pulse, there is negative charge deposition for $V_\mathrm{P}>0$ and positive charge deposition for $V_\mathrm{P}<0$, as seen between $t_\mathrm{s}=1087.5$ ns and $t_\mathrm{s}=1287.5$ ns. It also justifies the partial neutralization of surface charge at later stages, as is the case between $t_\mathrm{s}=1287.5$ ns and $t_\mathrm{s}=1812.5$ ns. As such, it is not obvious why the neutralization of the target surface is not completed in the timescale of a few $\upmu$s through electric drift and charge deposition. Furthermore, it is not clear yet why there are different surface charging memory effects according to $V_\mathrm{P}$. To shed some light on that question, simulation results of the spatial distributions of electric field magnitude ($E_\mathrm{t}$) and electron density ($n_\mathrm{e}$) in the plasma at different instants before and after the fall of the pulse of applied voltage are presented in Fig. 5, for both polarities of applied voltage and $|V_\mathrm{P}|=6$ kV. The same quantities are represented for $|V_\mathrm{P}|=5$ kV in Supplementary Fig. 5. The spatial distributions of simulated positive and negative ion densities are not shown, for the sake of brevity. Although there is attachment and charge separation, the density of negative ions does not overcome $10^{12}$ cm$^{-3}$ and, in the regions of high charge density (above $10^{12}$ cm$^{-3}$), the density of positive ions is approximately the same as $n_\mathrm{e}$.

Figure 5 reveals significant differences in structure and volume of the plasma plume between the different polarities after the fall of the pulse. With the fall of the pulse, there is an inversion of direction of the electric field, which afterwards is directed from the target to the plasma for $V_\mathrm{P}>0$ and from the plasma to the target for $V_\mathrm{P}<0$. As such, for positive polarity, the discharge acquires an expanding structure after the pulse, typical of negative discharges, due to outwards (from the plasma) electron drift. The opposite phenomenon takes place for negative polarity, acquiring a contracted structure, typical of positive discharges, due to inwards electron drift. For $V_\mathrm{P}=+6$ kV, we see in Fig. 4 that at $t_\mathrm{s}=1287.5$ ns, negative surface charge is deposited at the center, and positive surface charge is deposited on the edges, with maximum around $r=2$ mm. Then, in Fig. 5, we can notice that the plasma region adjacent to the target contains high electron densities at $t_\mathrm{s}=1200$ ns and at $t_\mathrm{s}=1500$ ns. In fact, at $t_\mathrm{s}=1500$ ns, 100 $\upmu$m above the target, $n_\mathrm{e}$ is higher than $10^{11}$ cm$^{-3}$ within a cross section of radius 1.46 mm. These high densities, together with the field generated by surface charges on the target, allow the plasma to provide charge fluxes to the surface that partially neutralize the target, as seen in Fig. 4 between $t_\mathrm{s}=1287.5$ ns and $t_\mathrm{s}=1812.5$ ns. Figure 5 shows that at later times (such as $t_\mathrm{s}=2500$ ns) this can no longer take place through electric drift, despite the presence of an electric field generated by surface charges, because charge densities in the plasma above the target are not significant. This is confirmed by the almost constant character of surface charge densities represented in Fig. 4 between $t_\mathrm{s}=1812.5$ ns and $t_\mathrm{s}=3412.5$ ns. Supplementary Fig. 5 shows that the same description is applied to $V_\mathrm{P}=+5$ kV, in which case the deposition region is smaller and the region where $n_\mathrm{e}$ is higher than $10^{11}$ cm$^{-3}$ at $t_\mathrm{s}=1600$ ns, 100 $\upmu$m above the target, has 1.0 mm radius.

For $V_\mathrm{P}=-6$ kV, Figs. 3 and 4 show that, at $t_\mathrm{s}=1287.5$ ns, positive charge is deposited at the center of the surface. Although the center is partially neutralized until $t_\mathrm{s}=3412.5$ ns, positive charge remains on the surface, with maximum density around $r=1$ mm. Figure 5 shows that this is related to free charge densities in the plasma plume, as the volume of high $n_\mathrm{e}$ above the target at $t_\mathrm{s}=1500$ ns is thinner than for $V_\mathrm{P}=+6$ kV and cannot cover the whole region of positive surface charge. In fact, 100 $\upmu$m above the target, the region where $n_\mathrm{e}$ is higher than $10^{11}$ cm$^{-3}$ at $t_\mathrm{s}=1500$ ns has only 1.2 mm radius (17% lower than 1.46 mm for $V_\mathrm{P}=+6$ kV). Following Fig. 2b and Supplementary Fig. 5, this feature is even more striking for $V_\mathrm{P}=-5$ kV, in which case charge densities in the plasma are low over the whole region adjacent to the target at $t_\mathrm{s}=1600$ ns, and thus positive surface charges cannot be neutralized, even at $r=0$. Indeed, 100 $\upmu$m above the target, the region where $n_\mathrm{e}$ is higher than $10^{11}$ cm$^{-3}$ at $t_\mathrm{s}=1600$ ns has only 0.7 mm radius (30% lower than 1.0 mm for $V_\mathrm{P}=+5$ kV). These results demonstrate the importance of discharge volume and structure, induced, among other parameters, by polarity and $V_\mathrm{P}$, for leftover surface charges and hence for the determination of memory effects.

## Conclusions

This work has directly analysed the axial component of electric field inside a dielectric target impinged by ionization waves (IWs) in a pulsed ($\sim ~1~\upmu$s on-time) plasma jet in different applied voltage conditions, by comparing simulations and experiments, yielding excellent agreement for every case. While positive polarity jets ($V_\mathrm{P}>0$) generate a star-shaped pattern of positive surface charge and electric field inside the target during the pulse, a more diffuse circular pattern of negative charge and electric field is induced during the pulse for negative polarity ($V_\mathrm{P}<0$). When the pulse falls there is a significant amount of surface charge deposition, mostly at the center of the target, of positive signal for $V_\mathrm{P}<0$ and of negative signal for $V_\mathrm{P}>0$. In longer time-scales, within 1 $\upmu$s after the fall of the pulse, the target is partially neutralized, but not totally. As such, counter-intuitively, when using $V_\mathrm{P}<0$, the target remains positively charged in between pulses ($200~\upmu$s off-time) in its central region. By showing this feature, this work uniquely demonstrates and quantifies leftover surface charges remaining on the target in long time-scales in between pulses. Moreover, the leftover surface charges have been shown to directly affect discharge dynamics, creating a charged cloud on top of the target surface as the IW approaches. The surface charging thus constitutes a relevant memory effect. As such, this article reveals the existence and importance of a fundamental feature of any plasma interacting with surfaces, with special relevance for dielectric barrier discharge operation.

It has been revealed that the amount of leftover surface charge density and related electric field is strongly dependent on $V_\mathrm{P}$. For $V_\mathrm{P}>0$, the leftover surface charge densities are not significant, reaching no more than $-10$ nC cm$^{-2}$, with associated field of $-1$ kV cm$^{-1}$. For $V_\mathrm{P}=-6$ kV, values as high as 20 nC cm$^{-2}$ and 2 kV cm$^{-1}$ remain, with maximum at $r=1$ mm. For $V_\mathrm{P}=-5$ kV, a centred distribution with maximum around 30 nC cm$^{-2}$ and 3 kV cm$^{-1}$ remains in the target in between pulses. The simulation results have indicated that these differences are due to the availability of charged particle densities in the plasma near the target that determines the possibility to neutralize the target. The plasma plume is more voluminous after the pulse for positive polarities, and thus provides sufficient charges to almost neutralize the target. For $V_\mathrm{P}=-6$ kV, the plasma plume after the pulse is thinner and, as such, can neutralize the target at $r=0$ but not at $r=1$ mm. Finally, for $V_\mathrm{P}=-5$ kV, the plasma plume is so depleted of charged particles at this stage that no significant surface neutralization takes place after the positive charging associated to the fall of the pulse.

## Methods

### Plasma jet configuration

**Figure 6:**
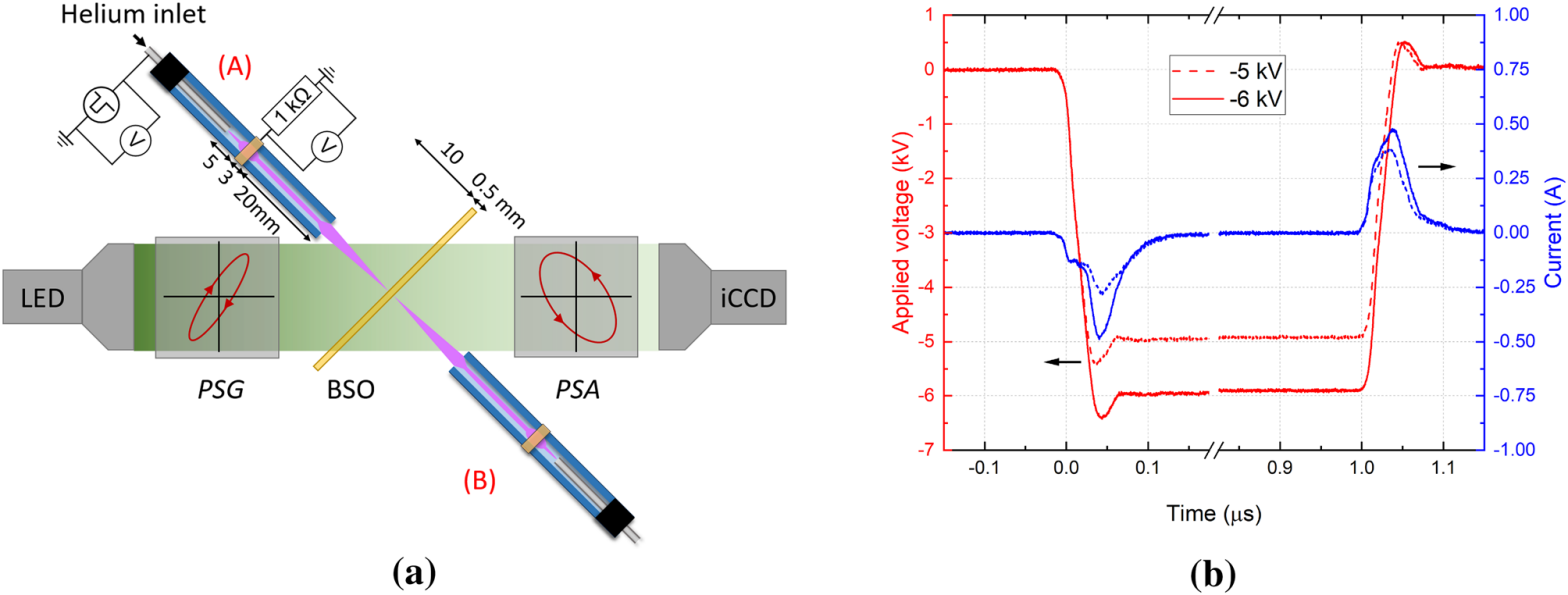
Experimental setup and voltage-current waveforms. (**a**) The experimental setup of the Mueller polarimeter used to measure the electric field inside the electro-optic BSO target under exposure of a negatively pulsed helium plasma jet positioned at either the front side (**A**) or back side (**B**) of the target. The z-axis is defined along the central axis of the jet, which implies that the target defines the XY-plane. (**b**) The waveforms of the applied voltage pulses (− 5 and − 6 kV) together with the resulting current measured over the 1 k$\Omega$ resistor at the outer ring electrode. Figure generated using Python 3.8^44^.

The plasma-target interaction is studied in this work using the same setup in experiments and simulations: a 5 kHz-pulsed atmospheric pressure plasma jet in coaxial configuration. The jet setup is schematically presented in Fig. 6, along with the experimentally observed temporal evolutions of current and applied voltage. The same jet system has been studied using rectangular pulses of applied voltage with positive polarity in Slikboer et al.^35^. A dielectric pyrex tube is used as part of the setup, with a relative permittivity of $\epsilon _\mathrm{r} = 4$, internal radius $r_\mathrm{in} = 1.25$ mm and outer radius $r_\mathrm{out} = 2.0$ mm. A dielectric BSO target with $\epsilon _\mathrm{r} = 56$ is placed perpendicularly to the tube at 1 cm from the end of the tube. The target is at a floating potential, has 0.5 mm thickness and is set between $z = 1.00$ cm and $z = 1.05$ cm (the end of the tube being placed at $z=0$). In the simulations, a grounded plane is set 10 cm behind the target.

Experimentally, an inner stainless steel tube is present within the pyrex capillary acting as powered electrode through which the helium flows into the capillary and then into the open air environment at 1 slm (*standard liter per minute*). The tip of the powered electrode is placed at 2.8 cm from the end of the tube. An outer grounded ring electrode of 3 mm length is present at 5 mm from the end of the powered electrode, leaving 2 cm till the end of the capillary. The same geometry is applied numerically, where the powered electrode is represented by a ring set inside the tube between $z = -2.8$ cm and $z = -3.3$ cm, with inner radius 0.4 mm and outer radius 1.25 mm. A pulse of positive or negative applied voltage is applied to the inner ring. The applied voltage increases in module from zero at $t_{0}=0$ ns during 50 ns, until reaching a plateau voltage $V_\mathrm{P}$. It is then constant until $t_\mathrm{f}$ and decreases in module for 50 ns, when it reaches zero, as in our previous works.

### Experimental setup

The quantification of surface charging by the IWs generated by the plasma jet is done using an advanced novel diagnostic called Mueller polarimetry. A similar setup has been used in previous works^33,48–50^ but the experimental procedure taken in this work, shown in Fig. 6, followed the configuration proposed in^34,35^. This optical technique allows for the examination of an electro-optic BSO material (Bi$_{12}$SiO$_{20}$) used as target in the plasma-surface interaction^51^. The refractive index of the material changes under external influences such as strain or the electric fields induced by surface charges following the Pockels effect. These changes cause the material to have a spatial and time-dependent birefringence, which has been detected and quantified with the Mueller polarimeter^52^. This includes the eigenvalue calibration method^53,54^ and the logarithmic decomposition to interpret the optical properties^55,56^.

The relation between the detected birefringence and the externally induced electric field depends on many factors, e.g. the materials properties (symmetry point group) and orientation with respect to the polarized light beam of the polarimeter^51^. As a result, the birefringence can be divided in various quantities of retardance. One component of the linear retardance, i.e. $\Gamma _{0/90}$, contains the relation with the external axial electric field $E_z$, as well as the horizontal component of the radial field $E_x$, according to

1 $$\begin{aligned} \Gamma _{0/90}= & {} \frac{2\pi d^*}{\lambda }\cdot n_o^3\cdot r_{41}\cdot \left( \sin (\theta )E_x - \cos (\theta ) E_z\right) , \end{aligned}$$

with electro-optic constant $r_{41}=4.8$ pm/V, wavelength $\lambda =550$ nm and refractive index $n_o=2.54$. The BSO material is examined at a 45$^\circ$ examination angle $\theta$ with respect to the Mueller polarimeter. Therefore the optical pathlength is $d^*=d/\cos (\theta )$ with thickness $d=0.5$ mm. This allows the plasma jet to interact with the target at normal incidence. Since an examination angle of 45$^\circ$ is followed, a similar procedure as proposed and used in^34,35^ is taken. This means a measurement is performed twice with the plasma jet either at the front side or on the backside of the target. This procedure allows to disentangle the electric field components from each other and from $\Gamma _{0/90}$, since only $E_z$ changes with sign when the location of the jet is reversed.

The Mueller polarimeter is designed to operate according to an external trigger event, which is related to the applied high voltage pulses used to generate the plasma. For each measurement 960 frames have been acquired (each at a different applied HV pulse) to obtain the characterization of the optical properties and thus the birefringence. As a result, a time-dependent image of the induced axial electric field strength is obtained revealing the location of surface charge deposited by the interaction of the ionization waves. Additionally, the light emission of the plasma is easily captured with the same system, allowing to compare the propagation of the IWs with the temporal dynamics of the surface charges.

A background is generally present within the birefringence relating to the temperature gradient along the material caused by (limited) heat transfer by the plasma^48,49^. This induces an internal strain that accumulates to the birefringence caused by the external electric field. Normally this background is subtracted by using a time-resolved measurement performed 100 $\upmu$s after the HV pulse has ended. This was possible in previous works because no leftover charges were found at this time and thus only the background was detected. Now, in this work, this did not apply because clear evidence of long term surface charges is observed. Therefore the background image due to temperature induced strain could not be removed. This is not a problem for the axial electric field since the temperature gradients did not change in sign when the plasma jet was moved to the other side of the targeted material. However, it is a problem for the radial components of electric field, which, therefore, are not included in this work.

### Numerical model

A two-dimensional axisymmetric fluid model is used to simulate the transient discharge dynamics. The model has been described in our previous works^35,36,43,57^. In particular, the same geometry is used as in Slikboer et al.^35^. The model assumes atmospheric pressure and room-temperature ($T = 300$ K) in the whole domain. In the experiments, helium flows through the tube with air impurities. The model considers helium flowing through the tube with 100 ppm of O$_2$ impurities and a 1 slm flow rate, as in the experimental conditions. Drift-diffusion-reaction equations are solved for mean electron energy, electrons, positive ions and negative ions, along with reaction equations for neutral species, coupled with Poisson’s equation in cylindrical coordinates (*z*, *r*). The kinetics in the He-O$_2$ plasma is described for a total of 10 species (e, O$_2^-$, He$^+$, He$_2^+$, O$_2^+$, He, He(2$^3$S,2$^1$S), O, O$_2$, O$_2$($a_1\Delta _g$)), using the reaction scheme proposed in Viegas and Bourdon^36^ and derived from Liu et al.^58^, including a total of 55 reactions.

At the surfaces, absorption of incoming electrons, recombination of positive ions (removing electrons from the surface) and detachment of negative ions (providing electrons to the surface) are considered with unit probability. Secondary electron emission is assumed to take place through positive ion impact. Unlike the case in our previous works, we consider this process not only for dielectric surfaces (tube and target) but also for metallic surfaces (inner electrode), with different emission coefficients, respectively, $\gamma _\mathrm{diel}=0.1$ and $\gamma _\mathrm{metal}=0.5$. It has been verified that the choice of $\gamma _\mathrm{metal}$ between 0.1 and 1 has a negligible influence on the discharge simulation results assessed in this work, i.e. in the plasma plume and in the dielectric target. Such high values of $\gamma _\mathrm{diel}$ and $\gamma _\mathrm{metal}$ are considered as an effective way to roughly take into account other secondary electron emission processes, such as through impact of excited species^59^, photoemission^60–62^, thermionic emission^63^ and field emission^63–65^. In fact, in the experimental study of Tschiersch et al.^66^, the effective secondary electron emission coefficients for different dielectric materials have been reported to be between 0.02 and 0.4. Values of that order have been calculated theoretically through solid-state considerations for dielectric surfaces^67^ and through quantum-kinetic methods for metallic surfaces^68^, as well as measured for metallic surfaces^69^. The surface charge density $\sigma$ on the surface of the dielectrics is obtained by integrating charged particle fluxes to the surface in time. These charges are then considered to remain immobile on the dielectric surface. Other works^70^ consider some conductivity inside dielectrics, but they intend to describe liquid surfaces or biological tissues.

As initial conditions, the model considers a standard uniform initial preionization density $n_\mathrm{init} = 10^9$ cm$^{-3}$ of electrons and O$_2^+$, as in our previous works. This assumption takes into account the high repetition rate in experiments ($f = 5$ kHz)^71^. As in our previous works, the plasma model has been coupled with static flow COMSOL calculations^34,72^. For a similar jet system without target in Hofmans et al.^57^, a comparison between flow calculations from the same model and radially-resolved Raman scattering measurements of air density (N$_2$ + O$_2$) has yielded a good agreement. Several model parameters are dependent on electron kinetics. These are calculated with the electron Boltzmann equation solver BOLSIG+^73^, using the IST-Lisbon database of cross sections in LXCat^74–76^, as functions of both the local mean electron energy $\epsilon _m$ and the local gas mixture. Photoionization is included in the model by using the approach described in^35,77^, that considers the ionizing radiation as being proportional to the excitation rate of helium atoms by electron impact.

The model takes a finite volume approach and a Cartesian mesh. The mesh size is 10 $\upmu$m, axially between $z = -3.3$ cm and $z = 1.05$ cm and in the radial direction between $r = 0$ and $r = 3.0$ mm. Then, the mesh size is expanded using a geometric progression until reaching the boundaries of the computational domain ($r = 10$ cm and $z = -10$ cm). As such, a mesh of n$_z$ $\times$ n$_r$ = 4400 $\times$ 370 = 1.628 million points is used. The results presented in this paper, obtained through 3.5 $\upmu$s simulation runs, require an average computational time of five days with 64 MPI processes on a multicore cluster “Hopper” (32 nodes DELL C6200 bi-pro with two 8-core processors, 64 GB of memory and 2.6 GHz frequency per node).

### Conditions for comparisons between experiments and simulations

As shown in the Results section, the experiments verify, through electric field measurements inside the BSO target, that surface charges can remain on the dielectric target surface in between pulses. Simulations lasting up to 2.5 $\upmu$s after the fall of the pulse confirm that surface charges remain on the dielectric target after the pulse, with constant density values from 2 $\upmu$s after the fall of the pulse, maximum density values as high as 30 nC cm$^{-2}$ and integrated values as low as -0.5 nC. Conversely, according to the simulation results, surface charge densities on the dielectric tube after the pulse are no higher in absolute value than 0.4 nC cm$^{-2}$. As such, to reproduce repetitive experimental conditions, the simulation for each applied voltage pulse condition is ran twice. The first time, the simulation is ran without initial surface charges, up to $t_\mathrm{s} = 3.5~\upmu$s (for $V_\mathrm{P}>0$, this is the same calculation as in Slikboer *et al.*^35^, except for the consideration of secondary electron emission from metallic surfaces). The surface charge density distribution on the target from the first run at $t_\mathrm{s} = 3.5~\upmu$s is taken as initial condition (at $t_\mathrm{s}=0$) for the second run, which is used for comparisons with experiments. As shown in the Results section, the initial surface charges can generate a charged cloud near the target during IW propagation. As such, what is meant by time of impact in this work in some cases refers to the instant in time when the propagating discharge connects with the charged cloud near the target.

As shown in the Results section, considering initial surface charges is a consistent approach, as the final surface charge density distribution simulated at $t_\mathrm{s} = 3.5~\upmu$s is approximately equal to the one taken as initial condition, and the corresponding electric field inside the BSO target agrees well with experimental measurements. However, the consideration of initial surface charges on the target does not guarantee a complete and accurate knowledge of initial conditions. Moreover, an O$_2$ atmosphere is considered in the simulations, instead of an air atmosphere. As a result of the uncertainty in initial conditions and gas mixture, the velocity of IW propagation in the plume and the time of discharge impact on the target in the simulations are different than those in the experiments. To compensate for the difference in time of impact, in this work, as in Slikboer et al.^35^, the rectangular pulses in the simulations are longer than in the experiments and the comparisons between experiments and simulations are performed for the same $V_\mathrm{P}$ and approximately the same charging time, i.e. the time from the impact at the surface until the fall of the high voltage pulse (starting at $t_\mathrm{f}$), rather than the same pulse duration. As a result, by shifting the simulation results in time, as in Slikboer et al.^35^, an excellent agreement between experimental and numerical results is obtained on the physics of discharge-target interaction, as shown in the Results section. In Tables 1 and 2 the different times of impact and pulse widths for the cases studied in this work are presented, together with the shift in time applied to simulation results when comparing them to the measurements and the total initial charge on the target in each simulation.

**Table 1:** Time of impact (ToI) of the discharge on the target in experiments for different $V_\mathrm{P}$.

$V_\mathrm{P}$ (kV)	$t_\mathrm{f}$ (ns)	ToI (ns)
− 5	1010	$380 \pm 20$
− 6	1010	$240 \pm 20$
+ 5	1010	$293 \pm 25$
+ 6	1010	$193 \pm 25$

**Table 2:** Time of impact (ToI$_\mathrm{s}$) and total initial charge ($Q_\mathrm{init}$) on the target in simulations for different $V_\mathrm{P}$ and $t_\mathrm{f}$.

$V_\mathrm{P}$ (kV)	$t_\mathrm{f}$ (ns)	ToI$_\mathrm{s}$ (ns)	Shift (ns)	$Q_\mathrm{init}$ (pC)
− 5	1200	$670 \pm 20$	260	− 459
− 6	1100	$410 \pm 20$	160	− 505
+ 5	1200	$480 \pm 10$	210	+ 70
+ 6	1100	$320 \pm 10$	160	+ 85

The pulse duration ($t_\mathrm{f}$) in simulations is extended to have a charging time comparable to the one in experiments. A temporal shift is applied to simulation results when comparing them with experiments.

## Supplementary information

Supplementary Figures.
