# Supplementary material for: Quantification of surface charging memory effect in ionization wave dynamics
Source: Sci Rep. 2022 Jan 21;12:1181. doi: 10.1038/s41598-022-04914-8 (PMC8782963; doi:10.1038/s41598-022-04914-8)
Supplement: Supplementary file 1 — Supplementary Figures. [file 41598_2022_4914_MOESM1_ESM.docx]

**Quantification of surface charging memory effect in ionization wave dynamics**

**Supplementary Information**

**Supplementary Discussion 1: Dynamics of ionization wave (IW) propagation during the pulse for |*V*_P_| = 5 kV.**

For |*V*_P_| = 5 kV (Supplementary Fig. 1), the same features are observed as for |*V*_P_| = 6 kV (Figure 1 in the main text), but the discharges are slower and thinner for lower |*V*_P_|, in agreement with literature. The influence of the surface charging memory effect on discharge dynamics in simulations is even clearer for *V*_P_ = -5 kV than for *V*_P_ = -6 kV, as the discharge impacts the target 60 ns later than in simulations without initial surface charge. This delay is attributed to the globally negative initial surface charging of -459 pC (see Table 2 in the main text).


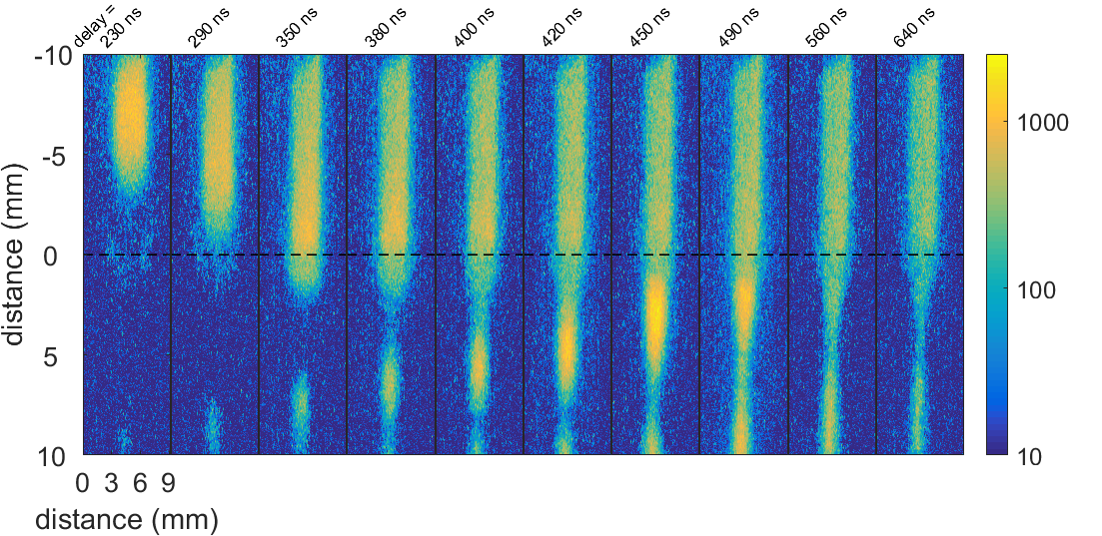


(a)


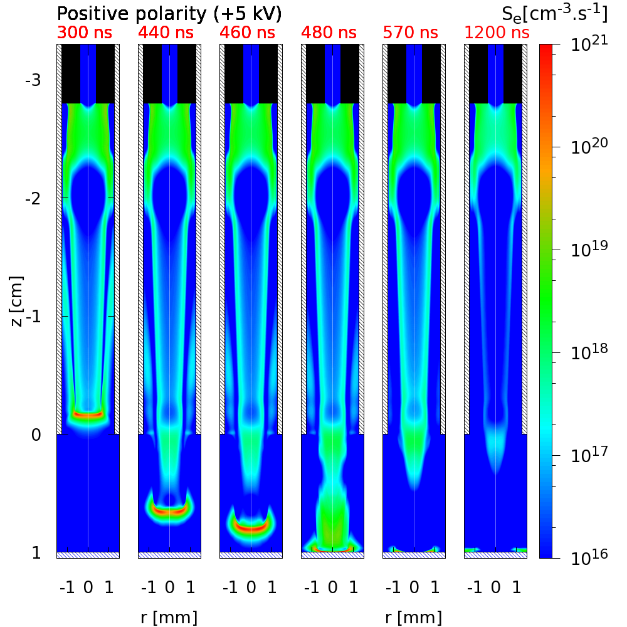

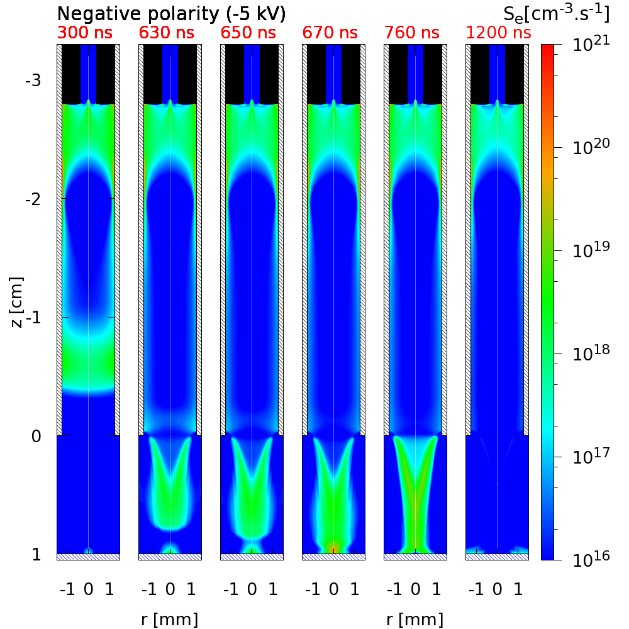


(b)

**Supplementary Figure 1: Dynamics of IW propagation during the pulse for |*V*_P_| = 5 kV.** **1(a):** Experimentally-obtained imaging of light emission at different instants during discharge propagation and interaction with the BSO target, for *V*_P_ = -5 kV. **1(b):** Spatial distribution of electron impact ionization source term (*S*_e_) from simulations, during discharge propagation and interaction with the BSO target, for |*V*_P_| = 5 kV and both polarities of applied voltage. The instants in time represented in Supplementary Fig. 1(b) refer to the time in simulations *t*_s_ and are not shifted.


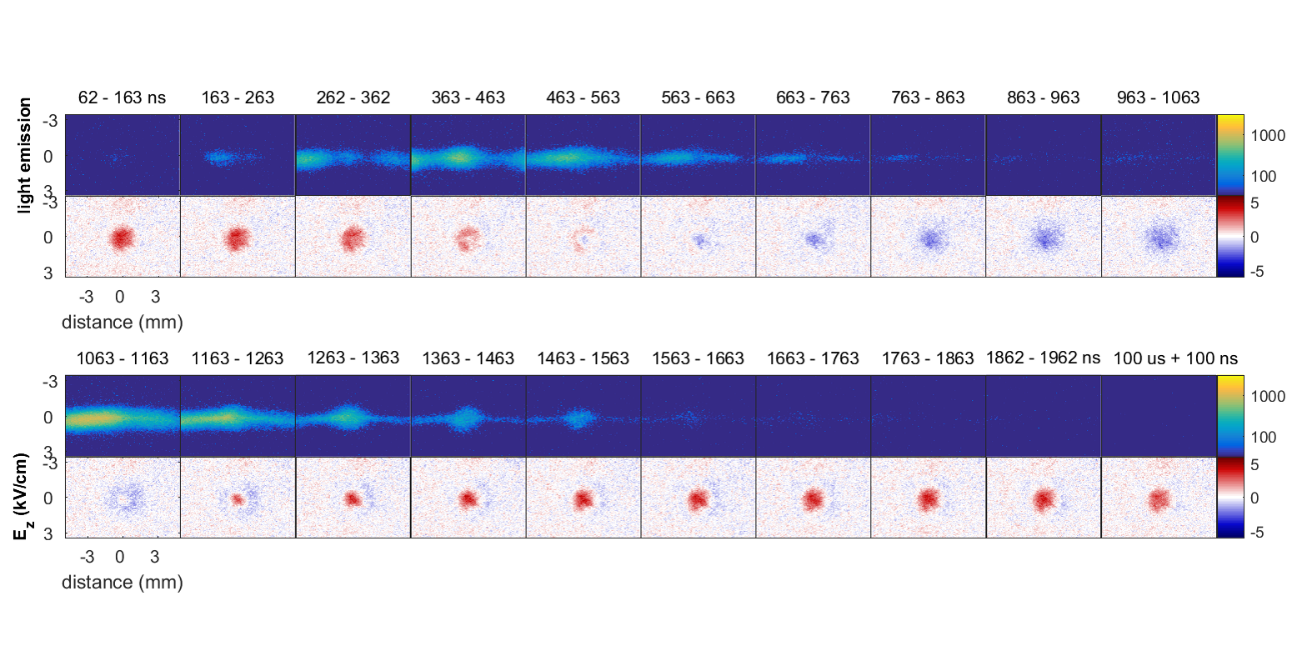


**Supplementary Figure 2: Experimental surface charging for |*V*_P_| = 5 kV.**

Measured light emission and axial electric field inside the BSO target at different instants, for *V*_P_ = -5 kV. 100 ns averaging is used.


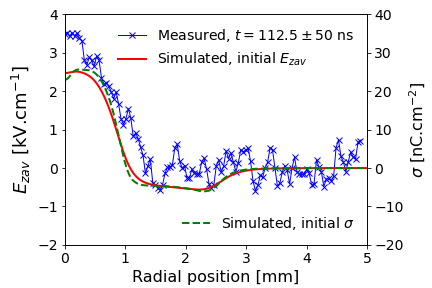

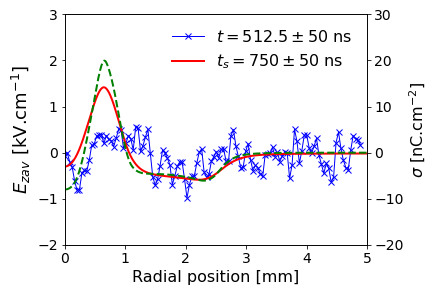

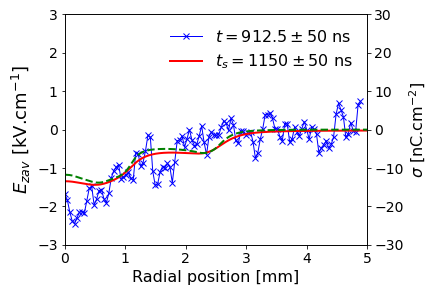

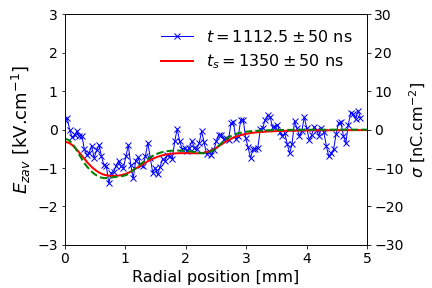

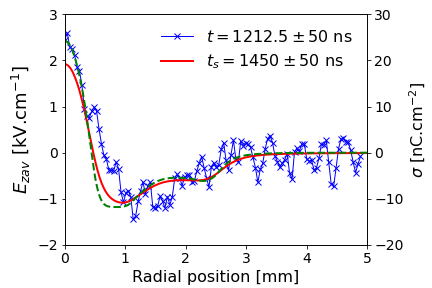

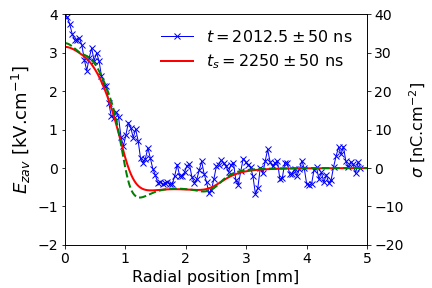


**Supplementary Figure 3: Radial profiles of surface charging for *V*_P_ = -5 kV.**

Radial profiles at different instants of the axial component of electric field inside the BSO target (*E_zav_*, solid lines) from simulations and measurements, and of the simulated surface charge density (σ, dashed lines) on the target surface, for *V*_P_ = -5 kV. 100 ns temporal averaging is used. *t* refers to the instant in time in experiments and *t*_s_ to the instant in time in simulations.


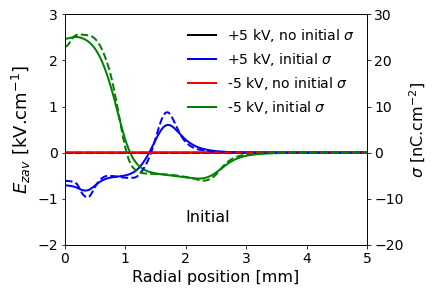

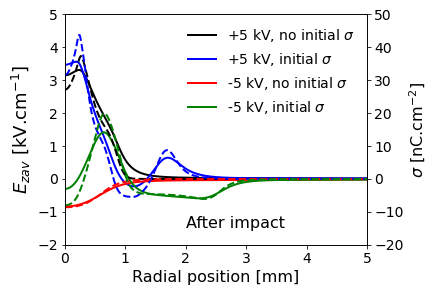

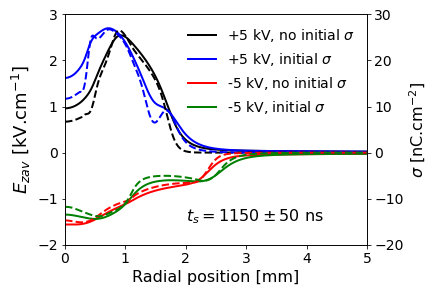

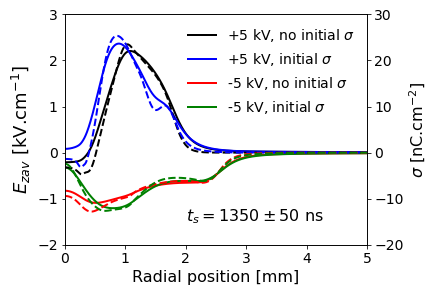

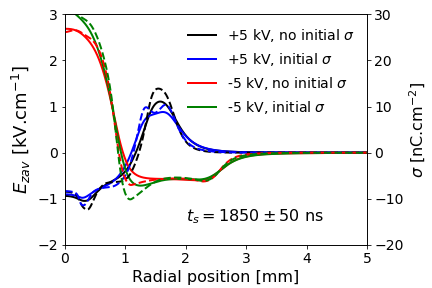

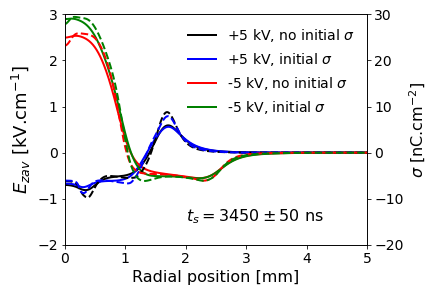


**Supplementary Figure 4: Simulated radial profiles of surface charging for |*V*_P_| = 5 kV.**

Radial profiles at different instants of the axial component of electric field inside the BSO target (*E_zav_*, solid lines) and of the surface charge density (σ, dashed lines) on the target surface, from simulations, for *V*_P_ = -5 kV and *V*_P_ = +5 kV, with and without considering initial surface charges. 100 ns temporal averaging is used.


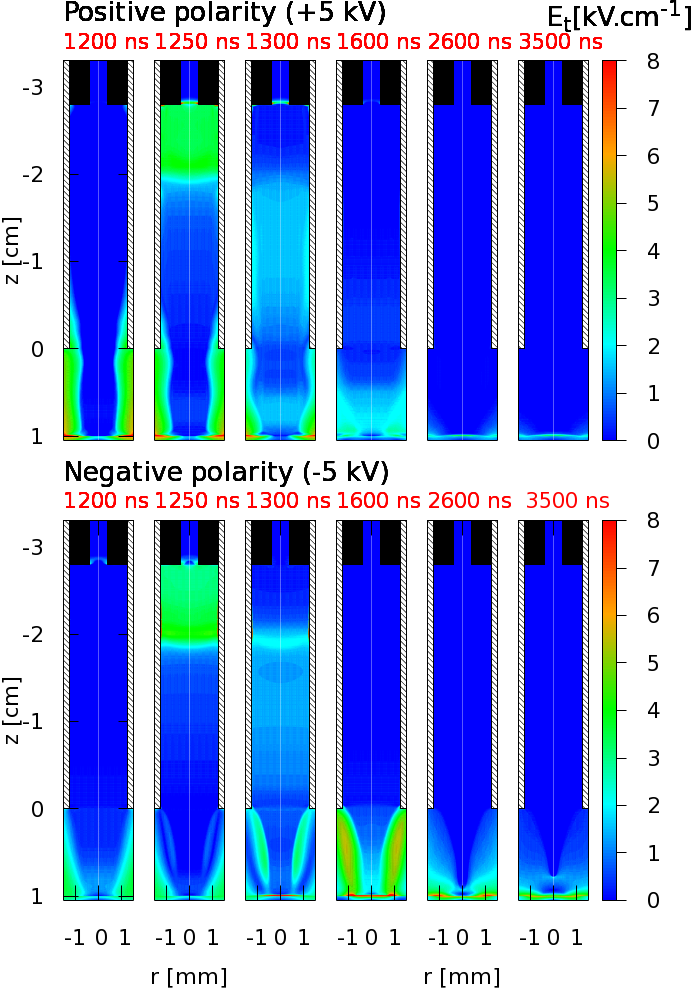

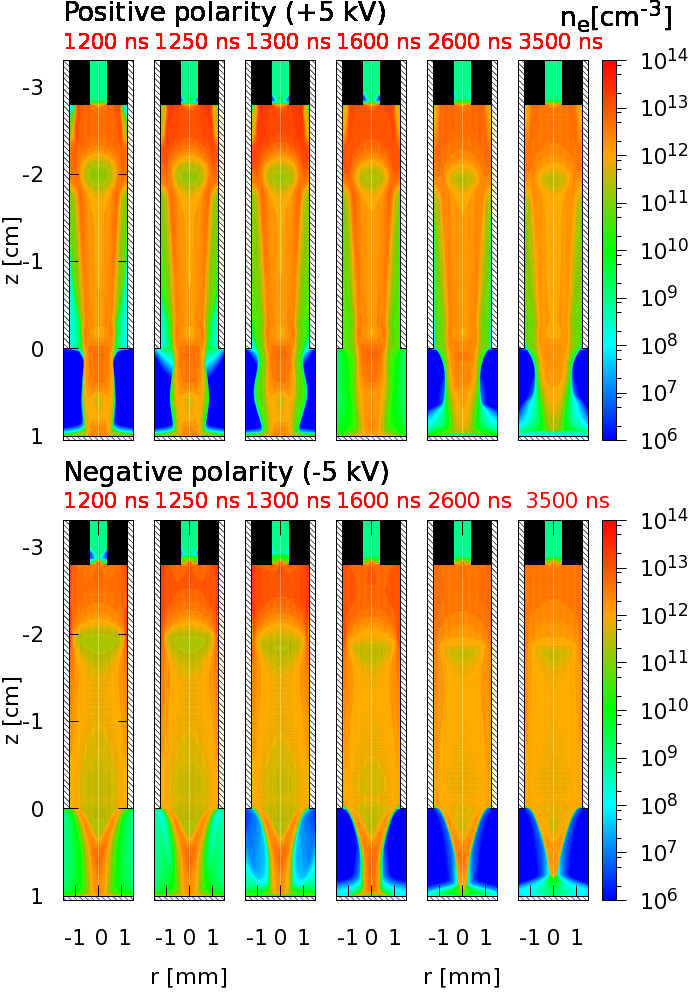


**Supplementary Figure 5: Simulated discharge dynamics after the pulse for |*V*_P_| = 5 kV.**

Electric field magnitude (*E*_t_) and electron density (*n*_e_) distributions from simulations, at the fall of the pulse, for |*V*_P_| = 5 kV and both polarities of applied voltage, at different instants *t*_s_.
